# Successful Treatment of Refractory IgA‐Mediated Autoimmune Hemolytic Anemia With Bortezomib

**DOI:** 10.1002/jha2.70162

**Published:** 2025-10-25

**Authors:** Silvia Neri, Corien L. Eckhardt, Boukje M. Beuger, Folman Folman, Eva Rettenbacher, Hanke L. Matlung, Taco W. Kuijpers, Josephine M. I. Vos, Robin van Bruggen

**Affiliations:** ^1^ Sanquin Research and Landsteiner Laboratory of the Academic Medical Center University of Amsterdam Amsterdam the Netherlands; ^2^ Department of Pediatric Hematology Location Academic Medical Center (AMC), Emma Children's Hospital, Amsterdam UMC, University of Amsterdam Amsterdam the Netherlands; ^3^ Department of Pediatric Infectious Disease Location Academic Medical Center (AMC), Emma Children's Hospital, Amsterdam UMC, University of Amsterdam Amsterdam the Netherlands; ^4^ Sanquin Diagnostic Services Amsterdam the Netherlands; ^5^ Department of Hematology Amsterdam UMC, University of Amsterdam Amsterdam the Netherlands

## Abstract

**Introduction:**

IgA‐mediated autoimmune hemolytic anemia (AIHA) is a rare condition associated with severe hemolysis and limited therapeutic response. Bortezomib, a proteasome inhibitor, targets plasma cells responsible for autoantibody production. Here, we describe a case of refractory IgA‐mediated AIHA in a 13‐year‐old boy presenting with severe hemolysis, who was successfully treated with bortezomib.

**Methods:**

Blood samples were collected at different time points throughout the disease course for immunohematology testing.

**Results:**

The patient showed significant hematologic improvement following four doses of Bortezomib with reduction in hemolysis and recovery of hemoglobin levels. Laboratory tests revealed complement‐negative, Coombs‐positive blood tests combined with altered RBC morphology. Phagocytosis of patient's RBC was absent at all timepoints. Notably, despite hematologic improvement, IgA‐positive RBC remained present, accompanied by compensated hemolysis.

**Conclusions:**

The present case demonstrates the potential of bortezomib as a treatment option for refractory AIHA cases, particularly in children.

**Trial Registration**: The authors have confirmed clinical trial registration is not needed for this submission

1

To the Editor,

Autoimmune hemolytic anemia (AIHA) is characterized by the destruction of red blood cells (RBCs) due to binding of autoantibodies against RBC epitopes [1, 2]. The majority of AIHAs are primarily mediated by IgG autoantibodies, sometimes accompanied by IgA or IgM, with or without complement activation [1, 2]. AIHA associated solely with IgA autoantibodies (pure IgA AIHA) is extremely rare [3, 4, 5, 6]. While the pathophysiology of IgG‐mediated AIHA typically involves Fc receptor‐mediated RBCs phagocytosis with or without complement activation, the mechanism of RBC destruction in IgA‐mediated AIHA, is not fully elucidated [1, 2, 5, 6, 7]. This lack of understanding complicates optimal treatment. IgA‐mediated AIHA can present with severe hemolysis and limited therapeutic response, posing significant challenges in clinical management [5, 8, 9, 10].

Bortezomib, a proteasome inhibitor used as anti‐plasma cell therapy, has been increasingly explored in the treatment of autoimmune cytopenias, including AIHA [11, 12, 13, 14]. Although most published evidence pertains to IgG‐mediated forms of AIHA, studies have shown encouraging responses with anti‐plasma cell therapies such as bortezomib and daratumumab in refractory AIHA cases [11, 12, 14]. Here, we report a case of a 13‐year‐old patient with severe refractory IgA‐mediated AIHA who achieved sustained transfusion‐independent clinical remission following treatment with bortezomib. However, morphological abnormalities persist, suggesting a low‐grade disease activity.

The patient (blood group A RhD positive, Ccee, K−), initially diagnosed with a mixed IgG‐ and IgA‐mediated AIHA in 2019, was admitted to the hospital with an episode of severe relapse (T1) in 2021 (Figure 1A, Table S1). Laboratory tests showed low hemoglobin (Hb), high lactate dehydrogenase (LDH), and elevated total bilirubin levels (Table S2). At relapse, predominantly IgA over IgG autoantibodies were detected by the direct antiglobulin test (DAT), both in the column and tube technique (Table S1). The patient responded well to steroid treatment in 2019, initiated at 2 mg/kg/day, reduced to 1 mg/kg after 1 week, and tapered off over the following 3 weeks. In contrast, the response was insufficient after relapse in April 2021. Steroids were restarted at 2 mg/kg/day (Figure 1A). Meanwhile, treatments with rituximab (four doses of 375 mg/m^2^, weekly) and sirolimus (target plasma level 6.0–10.0) were unsuccessful (Figure 1A).

**FIGURE 1:**
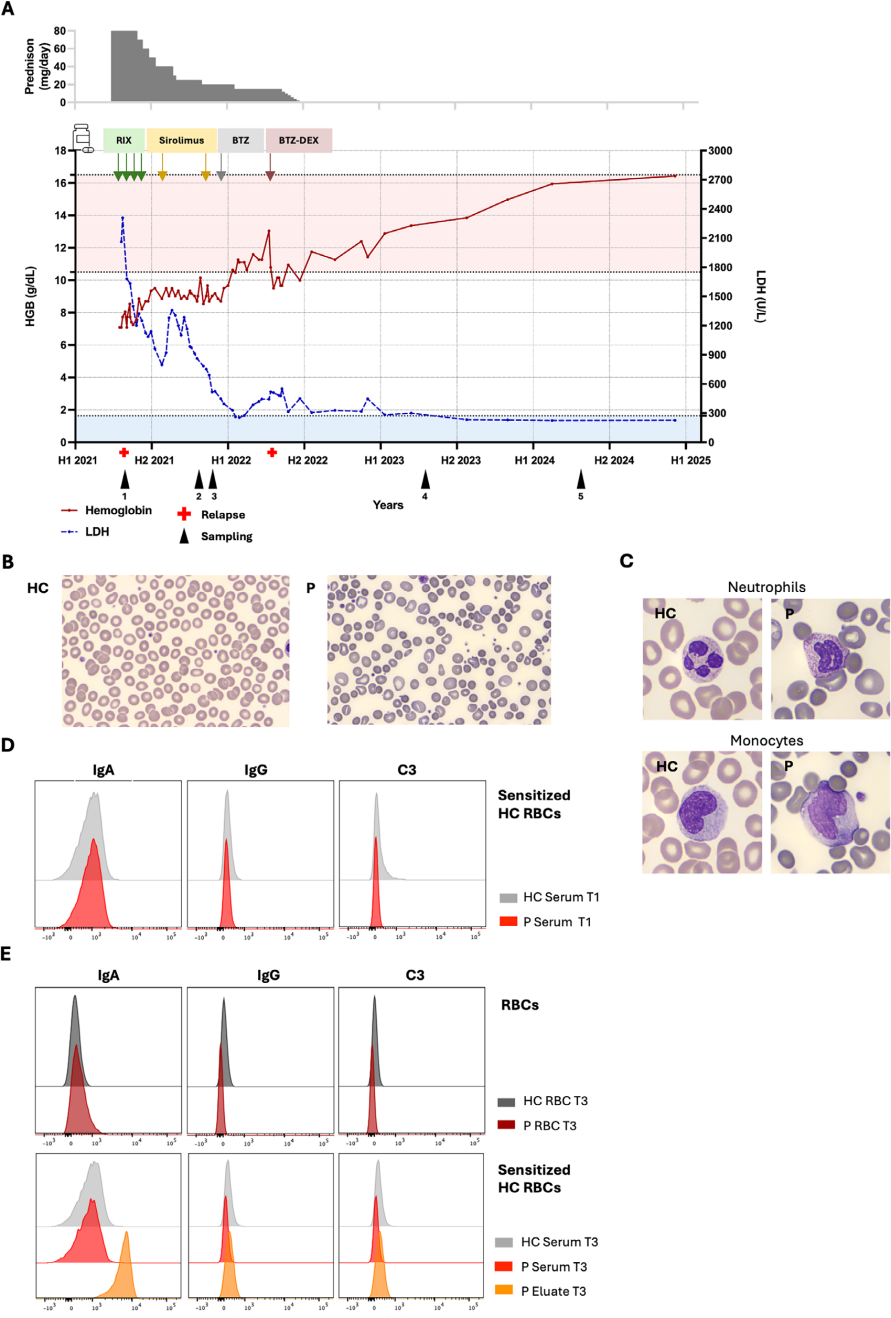
Clinical course, RBC morphology, and immunological features of IgA‐mediated AIHA during early disease and relapse. (A) Hemoglobin (g/dL; solid red line) and lactate dehydrogenase (LDH; IU/L; dotted blue line) levels over time. The graph shows treatments, episodes of relapse (red cross) and time of sampling (black arrows 1–5). H1 and H2 indicate first and second half of the year. (B) Peripheral blood smear at relapse (04‐2021; T1), showing marked RBC variability for the patient compared to healthy control. (C) Peripheral blood smear at relapse (T1) showing patient RBCs interacting with phagocytes compared to healthy control. (D) Flow cytometry analysis of IgA and IgG binding and complement deposition in healthy RBCs sensitized with patient serum at T1 compared to healthy control serum (*N *= 1). (E) Flow cytometry analysis of IgA and IgG binding and complement deposition in healthy and patient RBCs and in healthy control RBCs sensitised with healthy or patient serum and eluate at T3 (*N* = 1).

The high dose of corticosteroids could only be tapered slowly to 70 mg/day after 2 months from initiation in April, followed by 10 mg reductions every 2–4 weeks until 20 mg/day in October, and finally to 15 mg/day in January 2022, which was maintained until discontinuation in June 2022. This prolonged exposure led to significant side effects including weight gain > 20 kg, Cushing's syndrome, refractory hypertension, hypophosphatemia, hypokalemia, and osteopenia resulting in a spontaneous vertebral fracture. At T1, the peripheral blood smear revealed significant RBC morphological abnormalities such as spherocytosis and polychromasia, compared to healthy controls (Figure 1B). RBC‐phagocyte interactions were observed in the blood smear, suggesting potential FcR‐mediated RBC sequestration (Figure 1C). While the DAT test showed IgA on patient RBCs, healthy RBCs sensitized with patient serum showed no opsonization by IgA, IgG, and complement by flow cytometry (Figure 1D, Figure S1A). Phagocytosis of healthy RBCs sensitized with patient serum was assessed in vitro using neutrophils [15, 16]. Consistent with the flow cytometry results, no phagocytic uptake was observed for healthy RBCs sensitized with patient serum, comparable to those sensitized with healthy serum (Figure S2B,C). Due to limited sample availability, phagocytosis of patient's RBC could not be assessed at this time.

Six months after relapse (T2), patient RBCs still showed morphological alterations, such as microcytosis, hyperchromia and hypochromia, anisocytosis, and Hb concentration variation (Figure S1D,E). By the end of 2021 (T3), the patient's RBCs were opsonized with IgA and an eluate obtained from the patient's RBCs, strongly bound to healthy RBCs, while patient serum did not have this effect, suggesting very low level of circulating IgA antibodies and/or very high avidity (Figure 1E, left‐upper and lower panel; Figure S1F). IgG binding and complement deposition remained absent (Figure 1E, Figure S1F).

Abdominal ultrasound revealed progressive splenomegaly and hepatomegaly from relapse to the end of 2021. RBC scintigraphy indicated higher splenic RBC uptake than the liver (Figure S2A). Given the risk and complications of splenectomy, bortezomib was preferred as fourth‐line treatment. After ineffective treatment with rituximab and sirolimus, bortezomib (1.3 mg/m^2^/doses subcutaneously on Days 1, 4, 8, and 11) was initiated in December 2021, resulting in rapid normalization of RBC counts and other hematologic parameters (Figure 1A). In March 2022, the patient suffered from influenza A virus with high fever and gastroenteritis. An AIHA relapse followed in April 2022, with Hb at 9.5 g/dL, LDH two‐fold above the reference range and elevated reticulocyte count (Figure 1A, Table S2). A second cycle of bortezomib, combined with dexamethasone (three doses of 20 mg, weekly), again led to a swift clinical and partial biochemical response. The hemoglobin and LDH parameters normalized and the steroid therapy was discontinued (Figure 1A). The patient has remained in clinical remission since 3 years after the last bortezomib dose, with normal Hb and no further AIHA treatment needs. No bortezomib‐related side effects, including peripheral neuropathy, were reported.

Despite clinical improvement, in March 2023 (T4), blood smear still revealed significant RBC size heterogeneity (Figure S3A–C). IgA binding persisted on patient RBCs, but patient serum was unable to opsonize healthy RBCs (Figure S3D,E). In 2024 (T5), slight improvement in RBC morphology was observed compared to previous time points (Figure 2A,B). However, elevated RDW and persistence of few morphological alterations such as microcytosis, anisocytosis, hyperchromia, and Hb concentration variation, were still present, indicating incomplete recovery (Figure 2B,C). IgA was still bound to patient RBCs, without IgG binding or complement deposition, while no antibody binding occurred on healthy RBCs sensitized with patient serum (Figure 2D, Figure S4A). Although the patient RBC were IgA positive, phagocytosis of these cells was not induced in vitro. Similarly, the absence of phagocytosis was observed for the healthy RBCs sensitized with patient serum (Figure 2E, Figure S4B). This suggests that patient IgA autoantibodies may not efficiently induce FcR‐mediated phagocytosis, as similarly reported by another case [5, 7]. Nevertheless, blood smears at relapse (T1) showed RBC‐phagocyte interactions, suggesting possible FcαR‐mediated binding rather than uptake, potentially contributing to splenic congestion and hepatosplenomegaly. However, direct evidence for such a mechanism is lacking. The persistent IgA binding to patient RBC and RBC morphological alterations throughout the clinical course, suggests that the autoantibody may contribute to RBC membrane alteration and induce cellular damage, finally promoting hemolysis. This would align with the persistently and to date undetectable haptoglobin level (Table S2). His reticulocyte counts are progressively normalizing (peak of 793 per 10^6^/mL in September 2021 to 250 per 10^6^/mL in December 2024; Table S2), indicating a slow but steady improvement of his underlying AIHA.

**FIGURE 2:**
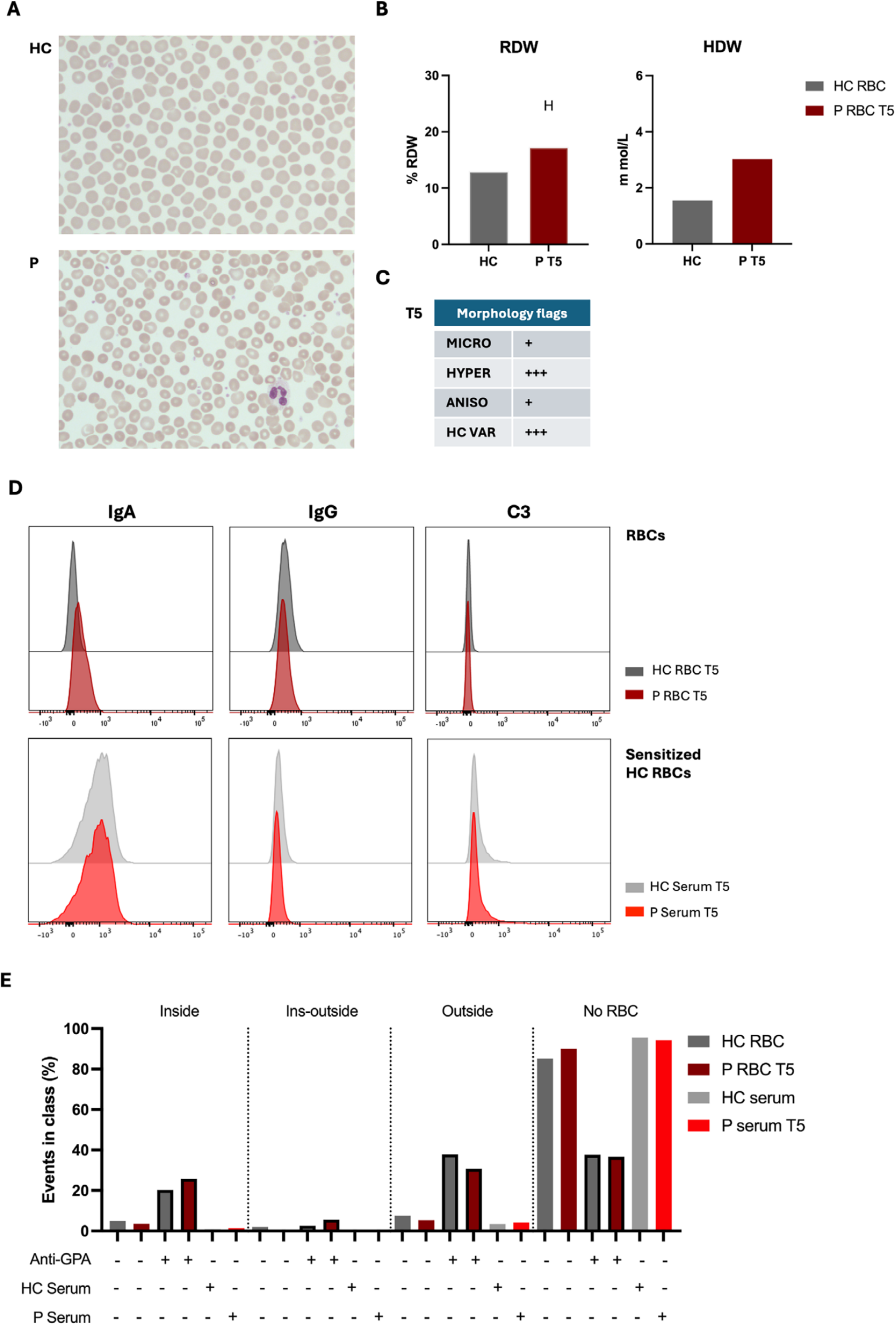
Comprehensive characterization of patient RBC morphology, antibody binding, and phagocytosis at T5. (A) Peripheral blood smear at T5 showing reduced variability of patient RBC morphology compared to previous time points. (B) RBC distribution width (RDW) and Hb distribution width (HDW) values of healthy and patient RBCs measured via ADVIA analyser at T5. Out‐of‐range values are marked as H (high) or L (low). (C) ADVIA morphology flags at T5 show high scores (3+) for hyperchromia (HYPER) and Hb concentration variation (HC VAR) in patient RBC. (D) Flow cytometry analysis of IgA and IgG binding and complement deposition in healthy and patient RBCs and in healthy RBC sensitised with healthy or patient serum at T5 (*N* = 1). (E) AI‐based IFC phagocytosis assay by neutrophils. The graphs show the percentage of events in classes. Opsonized RBCs with anti‐GPA antibody (Anti‐GPA) were used as positive control for phagocytosis. Phagocytosis was addressed in isolated patient RBCs compared to healthy control RBCs and healthy control RBCs sensitised with healthy or patient serum at T5 (*N* = 1).

In this case, the treatment with bortezomib lead to a swift and long‐term hematological response, supporting its role in reducing IgA autoantibody‐mediated RBC destruction.

The use of bortezomib also facilitated steroid tapering and finally cessation, which had already caused severe corticosteroid‐induced side effects, including Cushing's syndrome, osteoporosis, and multiple compression fractures of the patient's thoracic spine. Thus, bortezomib may represent an effective non‐cytotoxic option to treat refractory IgA‐mediated AIHA cases. Although bortezomib effectively mitigated the disease, the persistent IgA binding, RBC morphological abnormalities, undetectable haptoglobin level, elevated bilirubin, and reticulocytes count, alongside persisting hepatosplenomegaly, indicate incomplete disease resolution, raising concerns about potential future relapses. Serology data, obtained in 2023–2024, showed a lack of SARS‐CoV‐2 IgG antibody formation after vaccination, reflecting the impact of the rituximab treatment. In contrast, protective IgG against measles, mumps, and varicella persisted, suggesting preservation of long‐lived plasma cells, in line with the persistent detection of IgA against the patients’ RBCs. In addition, since April 2022, the traditional DAT has no longer been performed with antibody class differentiation, resulting in a negative outcome for antibody detection. However, flow cytometry consistently detected IgA on patient's RBCs throughout the disease course. This discrepancy highlights the importance of testing for specific antibody classes, particularly IgA, and the need for more sensitive diagnostic tools. Relying solely on standard DAT may underestimate the presence of IgA‐mediated hemolysis and delay appropriate intervention. Future research should focus on elucidating the specificity of IgA antibodies, their impact on RBC morphology, and exact mechanism of RBC destruction, as well as the long‐term outcomes of novel therapeutic strategies like bortezomib in AIHA cases.

## Author Contributions

S.N., C.L.E., J.M.I.V., and R.B. designed the study. S.N. and B.B. performed experiments. C.L.E., C.F., T.W.K., E.R., and J.M.I.V. collected clinical data and provided data interpretation. H.K. provided input on data interpretation. S.N. wrote the manuscript and designed figures. C.L.E., R.B., T.W.K., and J.M.I.V. reviewed and edited the manuscript. The final manuscript was reviewed and approved by all co‐authors.

## Ethics Statement

Ethical approval was obtained by the Sanquin Research institutional medical ethical committee, in accordance with the Declaration of Helsinki 2013.

## Consent

Patient and healthy donor blood samples were obtained after informed consent.

## Conflicts of Interest

J. M. I. Vos has received the following as institutional honoraria: research support from Beigene and AbbVie/Genmab; advisory board/consultancy fees from Sanofi and Janssen; and speaker fees from BMS, Sanofi, Beigene, Novartis, and Amgen; none are considered a conflict of interest for this manuscript. The other authors declare no conflicts of interest.

## Supporting information



Supporting File: jha270162‐sup‐0001‐SuppMat.docx

Supporting File: jha270162‐sup‐0002‐SuppMat.docx

**Table S1**: DAT results from Sanquin diagnostic laboratory. **Table S2**: Laboratory parameters at relapses and the latest check reported.**Figure S1**: (A) Flow cytometry analysis of IgA and IgG binding and complement deposition in healthy control RBC sensitised with patient serum and eluate at T=1. The gMFI of the fluorescent signal is shown (N=1). (B) AI‐based IFC phagocytosis assay by primary neutrophils. The graphs show the percentage of events in classes. Opsonized RBCs with anti‐GPA antibody (Anti‐GPA) were used as positive control for phagocytosis. Phagocytosis was addressed in healthy control RBCs sensitised with healthy or patient serum at T1 (N=1). (C) Representative image of events that fall into the ‘inside’ class for each condition of the assay after AI‐driven analysis using Amnis AI software. Hoechst+ neutrophils (cyan), DiD+ internalized erythrocyte (red). Images are shown in IDEAS software. (D) Red cell distribution width (RDW) and hemoglobin distribution width (HDW) of healthy and patient RBCs measured by an ADVIA hematology analyser at T2. Values considered out of the physiologic range are marked as H (high) or L (low). (E) Morphology flags detected in patient RBCs by a hematology analyser (T2). Several morphology flags were significantly present (score 3+) such as microcytosis (MICRO), anisocytosis (ANISO), hypochromia (HYPO), hyperchromia (HYPER) and hemoglobin concentration variation (HC VAR). (F) Flow cytometry analysis of IgA and IgG binding and complement deposition in healthy and patient RBC and in healthy RBC sensitised with patient serum and eluate at T3 by flow cytometry. Antibody signalling is shown (gMFI) (N=1).**Figure S2**: (A) RBC nuclear scan of the anterior and posterior abdomen of the patient after relapse (04‐2021) showing RBC uptake in the spleen. The scan was performed 1hr and 3 hr after injection of 99mTc‐labelled RBC. **Figure S3**: (A) Peripheral blood smears at T4, showing variability in RBC morphology compared to healthy control. (B) RDW and HDW of healthy and patient RBCs measured by an ADVIA hematology analyser at T4. Values considered out of the physiologic range are marked as H (high) or L (low). (C) Morphology flags detected in patient RBCs by ADVIA at T4. The morphological alterations present are microcytosis (MICRO, score 2+), anisocytosis (ANISO, score 2+), hyperchromia (HYPER, score 3+) and HC variation (HC VAR, score 3+). (D‐E) Flow cytometric detection of IgA binding in healthy donor and patient RBCs, and in healthy control RBCs sensitised with healthy control or patient serum at T4. Histogram (D) and gMFI of the fluorescent signal (E) are shown (N=1).**Figure S4**: (A) Flow cytometric detection of IgA and IgG binding complement deposition in healthy control and patient RBCs, and in healthy control RBCs sensitised with healthy control or patient serum at T5. The fluorescent signal (gMFI) is shown (N=1). (B) Representative image of events that fall into the ‘inside’ class for each condition of the assay after AI‐driven analysis using Amnis AI software. Hoechst+ neutrophils (cyan), DiD+ internalized erythrocyte (red). Images are shown in IDEAS software.

## Data Availability

Data sharing not applicable for this article.
