# Supplementary material for: Successful Treatment of Refractory IgA‐Mediated Autoimmune Hemolytic Anemia With Bortezomib
Source: EJHaem. 2025 Oct 25;6(6):e70162. doi: 10.1002/jha2.70162 (PMC12552891; doi:10.1002/jha2.70162)
Supplement: Supplementary file 1 — Supporting File: jha270162‐sup‐0001‐SuppMat.docx [file JHA2-6-e70162-s001.docx]

**Successful treatment of refractory IgA-mediated autoimmune hemolytic anemia with bortezomib**

**Supplemental material and methods**

**Sample collection**

Blood samples of the patient were collected at the Amsterdam University Medical Center (Amsterdam UMC), starting from the year 2019 when the patient was diagnosed with AIHA, until 2024. After informed consent, samples were sent to Sanquin reference centre for immunohematology diagnostics, while left over samples were either used fresh or stored at -80°C until use for research purposes. The time points of sampling for research purposes are indicated through the report as T1 to T5 (Figure 1A). Healthy donor blood samples were obtained after formal consent and were approved by the Sanquin Research institutional medical ethical committee, in accordance with the Declaration of Helsinki 2013.

**Immuno-hematological techniques**

The direct antiglobulin test (DAT) was performed in the diagnostic laboratory of Sanquin using both gel card (Bio-Rad, Diamed GmbH, Cressier, Switzerland) and tube technique with monospecific AHG sera (anti-IgG, anti-IgA, anti-IgM, anti-C3c, and anti-C3d, Essange Reagents, Amsterdam, The Netherlands). Eluate was prepared by acid elution using Gamma® ELU-KIT II® (Immucor, Inc., Norcross, GA, USA). All tests were performed according to the manufacturer’s protocol.

**ADVIA measurements and blood smears**

Blood cell counts and RBC phenotypic parameters were measured by an ADVIA 2120i hematology analyser (Siemens Healthineers, Erlangen, DE). Peripheral blood smears performed at Sanquin were made using 3 ul of whole blood and stained with May-Grünwald/Giemsa staining (Merck) to evaluate RBC morphology. Images were acquired using a Zeiss Axio Imager light microscope (Zeiss, Oberkochen, Germany) with a 50x objective.

**RBC scintigraphy**

RBC scintigraphy was performed using the semi *in-vivo* radiolabelling technique. The radiopharmaceutical 683 MBq Tc-99m-pertechnetate was used to label RBCs. 20 min after administration of 2 ml tin pyrophosphate, labelled RBC were injected into the patient (IV). The planar recordings were done at 1 and 3 hours p.i. of the upper abdomen.

**RBC and neutrophil isolation**

Cells were isolated from EDTA venous blood collected from healthy donors and the AIHA patient. Healthy and patient RBCs were separated from platelet-rich plasma and the peripheral blood mononuclear cells by centrifugation at 500g for 15 minutes. Platelet-rich plasma was collected and stored at -80°C until use. Subsequently, RBCs were washed twice with saline-adenine-glucose-mannitol (SAGM) buffer (150mM NaCl, 1.25mM adenine, 50mM glucose, 29mM mannitol; Fresenius Kabi, Bad Homburg, DE) and stored at 4°C until further use.^1^ Healthy neutrophils were isolated from whole blood by density-gradient centrifugation over isotonic Percoll (GE Healthcare, Chicago, USA) as previously described.^2^ The RBC pellet was lysed twice for 10 and 5 minutes with ice-cold isotonic NH_4_Cl solution (155 mmol/L NH_4_Cl,10 mmol/L KHCO_3_, 0.1 mmol/L EDTA, pH 7.4 (Merck, Darmstadt, DE)). Neutrophils were washed with phosphate-buffered saline (PBS) and resuspended in HEPES buffer (20mM HEPES, 132mM NaCl, 6mM KCl (Sigma-Aldrich, St. Louis, USA), 1mM MgSO_4_ and 1.2mM K_2_HPO_4_ (Merck) and supplemented with 0.5% (w/v) human serum albumin (HSA) (Brocacef, Maarssen, NL), 1mM CaCl_2_ (Calbiotech, El Cajon, USA), and 10mM glucose (Merck) (HEPES^+^ medium), and resuspended at a concentration of 5 x 10^6^ cells/mL and held at RT for functional studies.

**Flow cytometry**

To measure binding of antibodies and complement on isolated patient RBCs and in healthy donor RBCs treated with patient serum or eluate, IgA and IgG were detected using 10 µg/ml Goat Anti-Human IgA PE (Southern Biotech, Birmingham, USA) and 10 µg/ml Goat Anti-Human IgG (H&L) AF-488 (Invitrogen). Complement deposition was detected with 10 µg/ml mouse anti-human C3-19 (Sanquin, The Netherlands) primary antibody and incubated with 10 µg/ml Goat Anti-Mouse IgG (H&L) AF488 (Invitrogen) secondary antibody. Samples were analyzed on an LSRII or Canto II flow cytometers (BD Biosciences, San Jose, CA, USA) and data analysis was performed using FlowJo software v10 (BD Biosciences).

**AI-based imaging flow cytometry phagocytosis assay**

Biotinylation of 1 x 10^8^ RBCs was performed at 37°C for 30 minutes with 30 µg/ml biotin (EZ-Link™ Sulfo-NHS-LC-Biotin; Thermo Fisher Scientific, Waltham, United States) in PBS. After washing, RBCs were resuspended in 1 ml and stained with DiD (1,1′-dioctadecyl-3,3,3′, 3′-tetramethylindodicarbocyanine 4-chlorobenzenesulfonate salt) lipophilic membrane dye (Life Technologies, Carlsbad, USA), diluted at 2 μM, at 37°C for 20 minutes. Next, healthy and patient RBCs were either opsonized with 20 µg/ml anti-GPA (CD235a Monoclonal Antibody clone YTH89.1; Invitrogen, Waltham, USA) as positive control for phagocytosis at RT for 30 minutes or left non-opsonized. To sensitize healthy RBCs, cells were incubated at 37°C for 30 minutes with full patient serum and the binding of IgA, IgG antibodies and C3 deposition in sensitized RBCs was measured by LSRII flow cytometers (BD Biosciences). After isolation, 5 × 10^6^ neutrophils/mL were stained using Hoechst 33342 cell-permeant dye (Thermo Fisher Scientific) prior phagocytosis for 30 minutes at RT, to stain cell nuclei. RBCs were incubated with neutrophils at 37°C for 45 minutes in a U-bottom 96-well plate (Corning, Corning, USA) (ratio 1:2.5, neutrophil:erythrocytes). At the end of the assay, cells were centrifuged at 4°C at 500 g for 5 minutes and placed on ice to stop the phagocytosis. Afterwards, samples were stained with 10 µg/ml Streptavidin Alexa Fluor 488 conjugate (Thermo Fisher Scientific) at 4°C for 30 minutes. Finally, cells were fixed with 3.7% paraformaldehyde (PFA) at 4°C for 10 minutes and washed with ice-cold PBS. Erythrophagocytosis was measured by imaging flow cytometry (IFC; Image Stream X Mk II, Cytek Bioscences, Fremont, United States) and analyzed with IDEAS 6.3 software and Amnis Artificial Intelligence (AI) software v. 2.0.7 (Cytek Bioscences) as described previously.^3^

**References**

1. Burger, P., Hilarius-Stokman, P., De Korte, D., Van Den Berg, T. K. & Van Bruggen, R. CD47 functions as a molecular switch for erythrocyte phagocytosis. (2012) doi:10.1182/blood-2011.

2. Kuijpers, T. W. *et al.* Membrane surface antigen expression on neutrophils: a reappraisal of the use of surface markers for neutrophil activation. *Blood* 78, 1105–1111 (1991).

3. Neri, S. *et al.* An AI‐based imaging flow cytometry approach to study erythrophagocytosis. *Cytometry Part A* 105, 763–771 (2024).
