# Supplementary material for: Successful Treatment of Refractory IgA‐Mediated Autoimmune Hemolytic Anemia With Bortezomib
Source: EJHaem. 2025 Oct 25;6(6):e70162. doi: 10.1002/jha2.70162 (PMC12552891; doi:10.1002/jha2.70162)
Supplement: Supplementary file 2 — Supporting File: jha270162‐sup‐0002‐SuppMat.docx [file JHA2-6-e70162-s003.docx]

**Successful treatment of refractory IgA-mediated autoimmune hemolytic anemia with bortezomib**

**Supplementary Data**

**
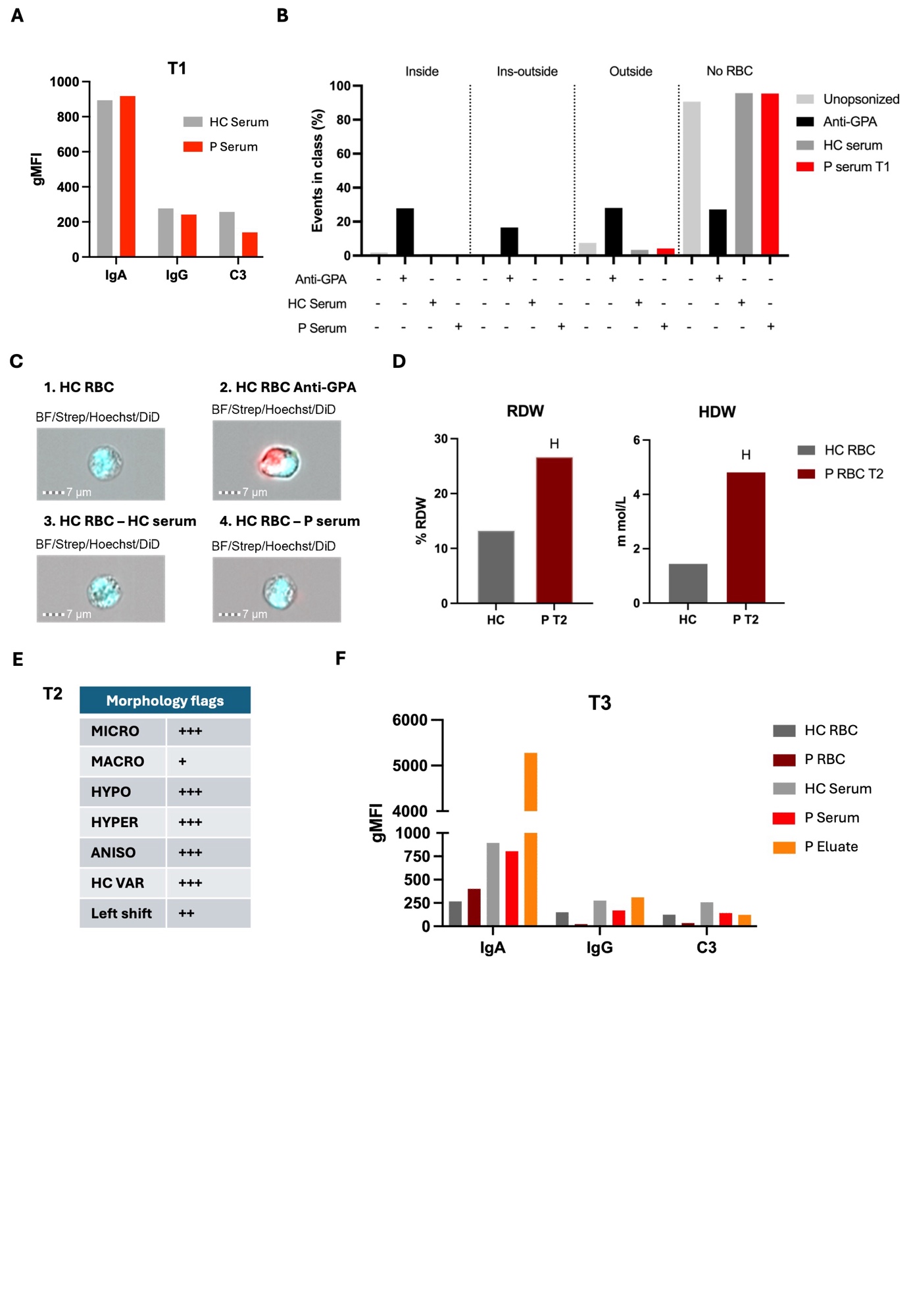
**

**Figure S1.** (A) Flow cytometry analysis of IgA and IgG binding and complement deposition in healthy control RBC sensitised with patient serum and eluate at T=1. The gMFI of the fluorescent signal is shown (N=1). (B) AI-based IFC phagocytosis assay by primary neutrophils. The graphs show the percentage of events in classes. Opsonized RBCs with anti-GPA antibody (Anti-GPA) were used as positive control for phagocytosis. Phagocytosis was addressed in healthy control RBCs sensitised with healthy or patient serum at T1 (N=1). (C) Representative image of events that fall into the ‘inside’ class for each condition of the assay after AI-driven analysis using Amnis AI software. Hoechst^+^ neutrophils (cyan), DiD^+^ internalized erythrocyte (red). Images are shown in IDEAS software. (D) Red cell distribution width (RDW) and hemoglobin distribution width (HDW) of healthy and patient RBCs measured by an ADVIA hematology analyser at T2. Values considered out of the physiologic range are marked as H (high) or L (low). (E) Morphology flags detected in patient RBCs by a hematology analyser (T2). Several morphology flags were significantly present (score 3+) such as microcytosis (MICRO), anisocytosis (ANISO), hypochromia (HYPO), hyperchromia (HYPER) and hemoglobin concentration variation (HC VAR). (F) Flow cytometry analysis of IgA and IgG binding and complement deposition in healthy and patient RBC and in healthy RBC sensitised with patient serum and eluate at T3 by flow cytometry. Antibody signalling is shown (gMFI) (N=1).

**
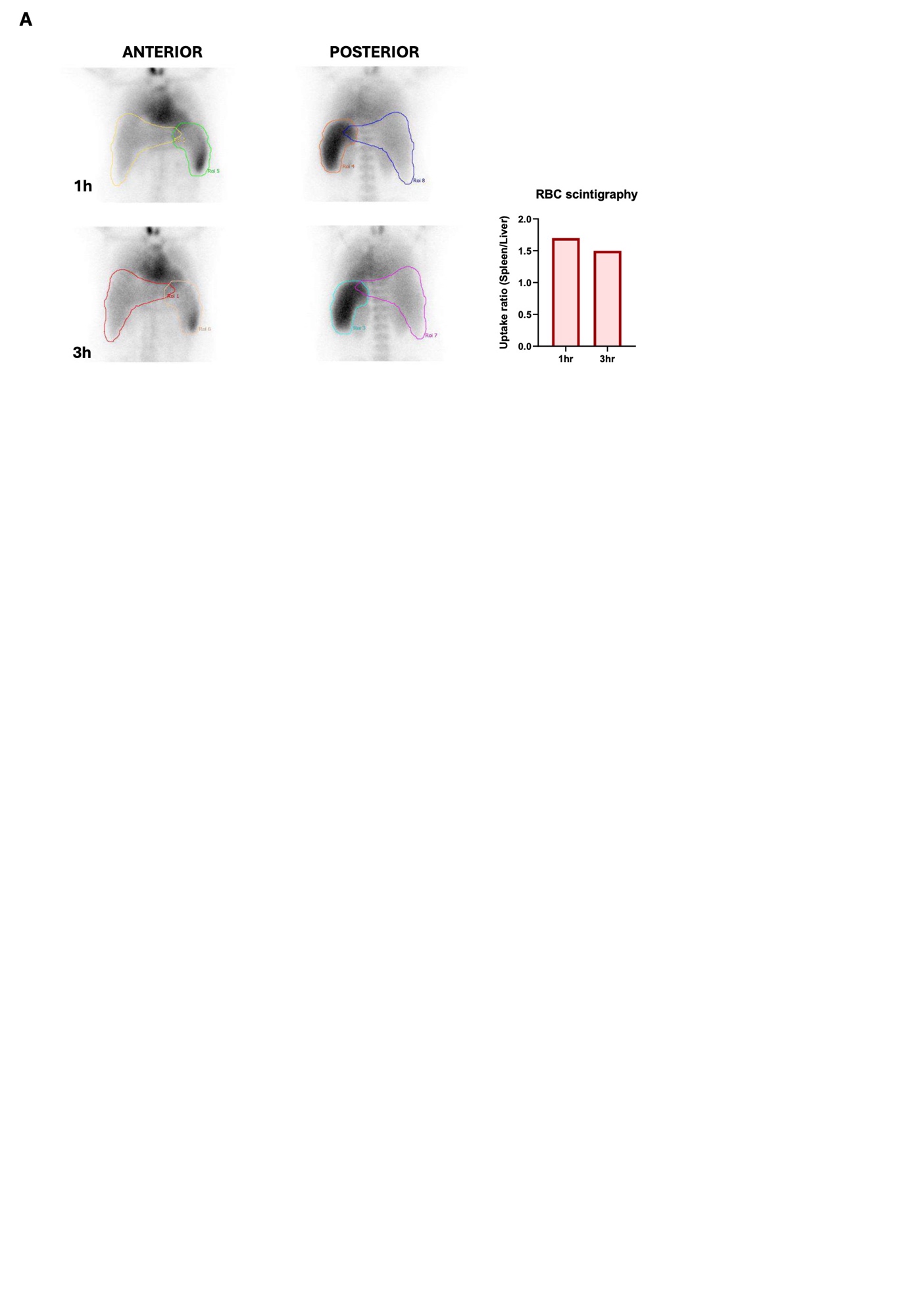
**

**Figure S2.** (A) RBC nuclear scan of the anterior and posterior abdomen of the patient after relapse (04-2021) showing RBC uptake in the spleen. The scan was performed 1hr and 3 hr after injection of ^99m^Tc-labelled RBC.


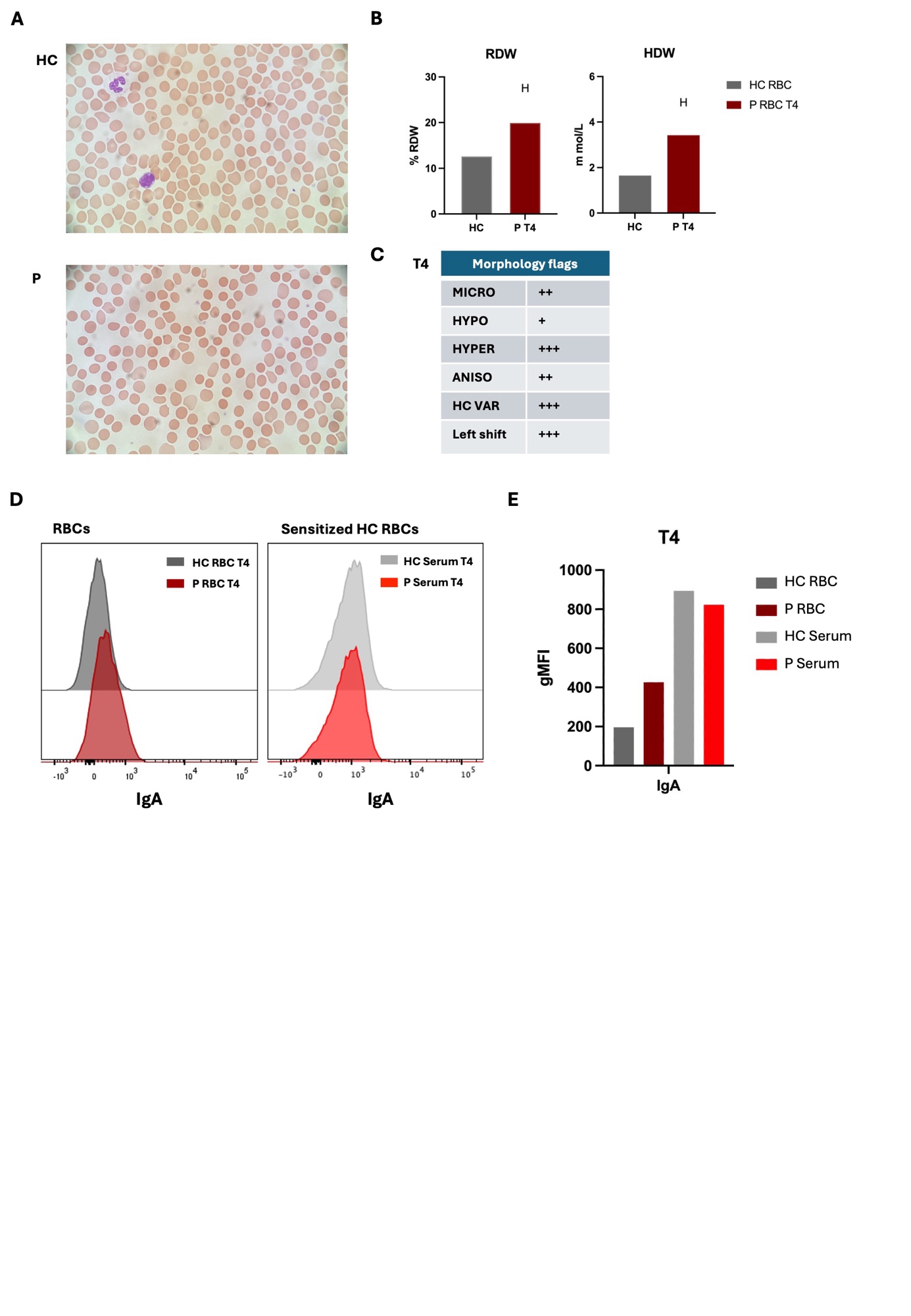


**Figure S3.** (A) Peripheral blood smears at T4, showing variability in RBC morphology compared to healthy control. (B) RDW and HDW of healthy and patient RBCs measured by an ADVIA hematology analyser at T4. Values considered out of the physiologic range are marked as H (high) or L (low). (C) Morphology flags detected in patient RBCs by ADVIA at T4. The morphological alterations present are microcytosis (MICRO, score 2+), anisocytosis (ANISO, score 2+), hyperchromia (HYPER, score 3+) and HC variation (HC VAR, score 3+). (D-E) Flow cytometric detection of IgA binding in healthy donor and patient RBCs, and in healthy control RBCs sensitised with healthy control or patient serum at T4. Histogram (D) and gMFI of the fluorescent signal (E) are shown (N=1).

**
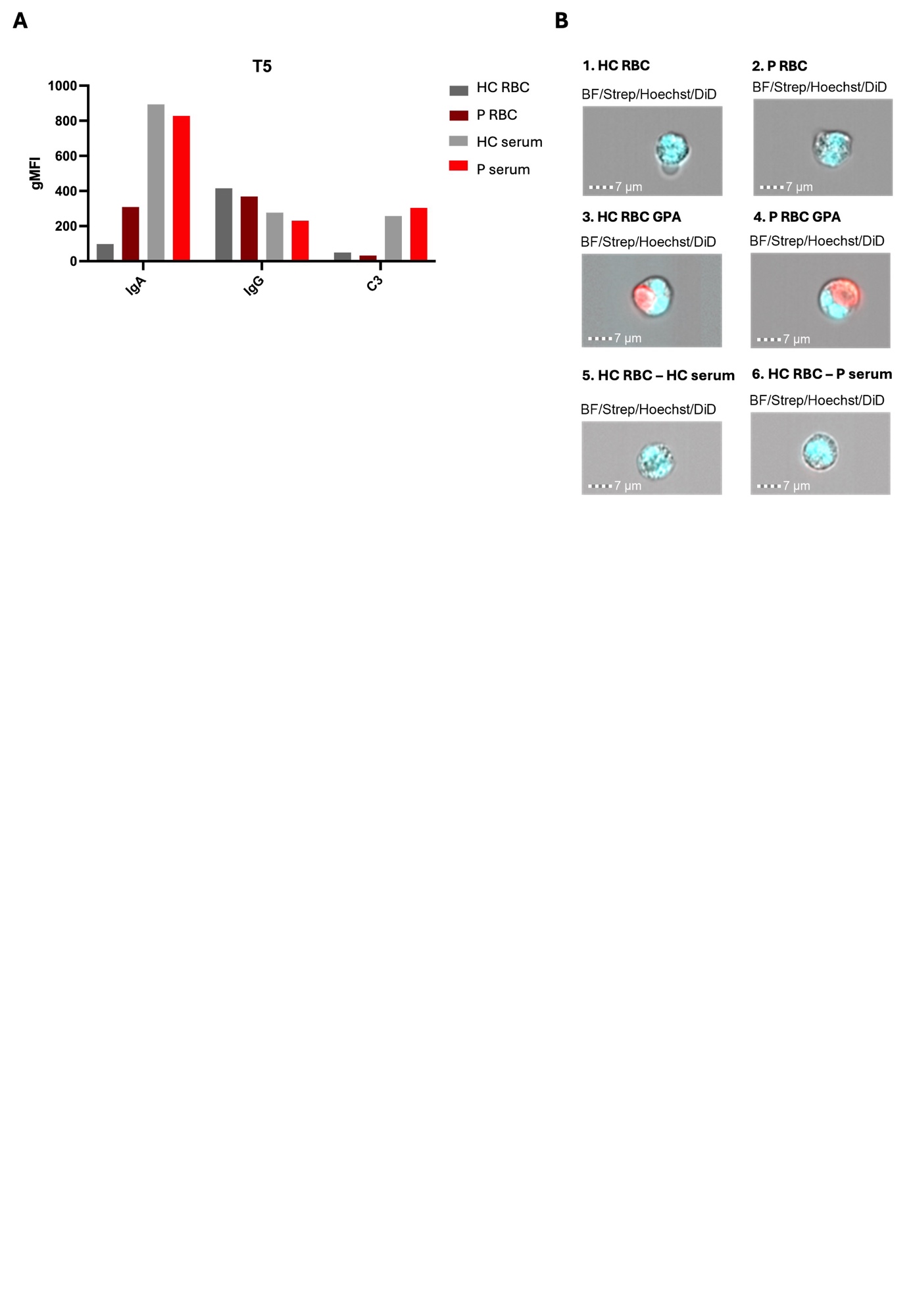
**

**Figure S4.** (A) Flow cytometric detection of IgA and IgG binding complement deposition in healthy control and patient RBCs, and in healthy control RBCs sensitised with healthy control or patient serum at T5. The fluorescent signal (gMFI) is shown (N=1). (B) Representative image of events that fall into the ‘inside’ class for each condition of the assay after AI-driven analysis using Amnis AI software. Hoechst^+^ neutrophils (cyan), DiD^+^ internalized erythrocyte (red). Images are shown in IDEAS software.
