# Supplementary material for: Successful Treatment of Refractory IgA‐Mediated Autoimmune Hemolytic Anemia With Bortezomib
Source: EJHaem. 2025 Oct 25;6(6):e70162. doi: 10.1002/jha2.70162 (PMC12552891; doi:10.1002/jha2.70162)
Supplement: Supplementary file 3 — Table S1: DAT results from Sanquin diagnostic laboratory. Table S2: Laboratory parameters at relapses and the latest check reported.Figure S1: (A) Flow cytometry analysis of IgA and IgG binding and complement deposition in healthy control RBC sensitised with patient serum and eluate at T=1. The gMFI of the fluorescent signal is shown (N=1). (B) AI‐based IFC phagocytosis assay by primary neutrophils. The graphs show the percentage of events in classes. Opsonized RBCs with anti‐GPA antibody (Anti‐GPA) were used as positive control for phagocytosis. Phagocytosis was addressed in healthy control RBCs sensitised with healthy or patient serum at T1 (N=1). (C) Representative image of events that fall into the ‘inside’ class for each condition of the assay after AI‐driven analysis using Amnis AI software. Hoechst+ neutrophils (cyan), DiD+ internalized erythrocyte (red). Images are shown in IDEAS software. (D) Red cell distribution width (RDW) and hemoglobin distribution width (HDW) of healthy and patient RBCs measured by an ADVIA hematology analyser at T2. Values considered out of the physiologic range are marked as H (high) or L (low). (E) Morphology flags detected in patient RBCs by a hematology analyser (T2). Several morphology flags were significantly present (score 3+) such as microcytosis (MICRO), anisocytosis (ANISO), hypochromia (HYPO), hyperchromia (HYPER) and hemoglobin concentration variation (HC VAR). (F) Flow cytometry analysis of IgA and IgG binding and complement deposition in healthy and patient RBC and in healthy RBC sensitised with patient serum and eluate at T3 by flow cytometry. Antibody signalling is shown (gMFI) (N=1).Figure S2: (A) RBC nuclear scan of the anterior and posterior abdomen of the patient after relapse (04‐2021) showing RBC uptake in the spleen. The scan was performed 1hr and 3 hr after injection of 99mTc‐labelled RBC. Figure S3: (A) Peripheral blood smears at T4, showing variability in RBC morphology compared to healthy control. (B) RDW a [file JHA2-6-e70162-s002.docx]

**Successful treatment of refractory IgA-mediated autoimmune hemolytic anemia with bortezomib**

**Supplementary Tables**

| Date | 10-2019 | 04-2021  T1 | 10-2021  T2 | 12-2021  T3 |
| --- | --- | --- | --- | --- |
| DAT Column | IgG 3+/IgA 2+ | IgG 2+/IgA 3+ | IgG neg/IgA 2+ | IgG neg/IgA 3+ |
| DAT Tubes | IgG 2+/IgA + | IgA weak + | IgA weak + | IgA 1+ |
| Eluate | Auto-e  Aspecific IgG>IgA | Aspecific IgG<IgA | Aspecific IgG<IgA | Aspecific IgA |
| Plasma | Auto-e of the IgG and IgA class | Auto-e | Neg | Neg |

**Table S1:** DAT results from Sanquin diagnostic laboratory.

| PARAMETERS | 1st Relapse  (04-2021) | 2nd Relapse (04-2022) | Latest Check  (12-2024) | Reference Value |
| --- | --- | --- | --- | --- |
| Hemoglobin | 4.4 | 5.9 | 16.4 | 10.5-16.5 g/dL |
| Thrombocytes | 306 | 306 | 212 | 150-450 x10^9/L |
| Reticulocytes | - | 519.50 | 250 | 35-105 x10^9/L |
| Haptoglobin | - | - | <0.10 | 0.30-2.00 g/L |
| Bilirubin (Total) | 130 | 53 | 59 | 4-29 µmol/L |
| LDH | 2062 | 510 | 226 | <273 U/L |

**Table S2:** Laboratory parameters at relapses and the latest check reported.
